# Nationwide Epidemiology and Management Time Trends for Atrial Fibrillation: Insights From the Korean AF Factsheet

**DOI:** 10.1016/j.jacasi.2025.03.012

**Published:** 2025-05-27

**Authors:** So-Ryoung Lee, Daehoon Kim, Yun Gi Kim, Pil-Sung Yang, Ki Hong Lee, Jaemin Shim, Bong-Seong Kim, Kyung-Do Han, Eue-Keun Choi

**Affiliations:** a Department of Internal Medicine, Seoul National University Hospital, Seoul, Republic of Korea; b Department of Internal Medicine, Seoul National University College of Medicine, Seoul, Republic of Korea; c Division of Cardiology, Department of Internal Medicine, Severance Cardiovascular Hospital, Yonsei University College of Medicine, Seoul, Republic of Korea; d Division of Cardiology, Department of Internal Medicine, Korea University College of Medicine and Korea University Anam Hospital, Seoul, Republic of Korea; e Division of Cardiology, Department of Internal Medicine, CHA Bundang Medical Center, CHA University, Seongnam, Republic of Korea; f Division of Cardiology, Department of Internal Medicine, Chonnam National University Medical School and Hospital, Gwangju, Republic of Korea; g Statistics and Actuarial Science, Soongsil University, Seoul, Republic of Korea

**Keywords:** anticoagulation, atrial fibrillation, epidemiology, prevalence, rhythm control

## Abstract

Atrial fibrillation (AF) represents the most prevalent cardiac arrhythmia in clinical practice, with its incidence rising globally. Korea's comprehensive national health insurance system facilitates the meticulous collection and management of health care utilization data for its entire population. This robust data infrastructure has enabled numerous recent studies on AF in Korea, encompassing its prevalence, incidence, anticoagulation treatment rates, health care burden, and associated complications. A comprehensive understanding of AF epidemiology and patient characteristics is essential for enhancing both primary and secondary prevention strategies and improving clinical outcomes. This review presents an up-to-date analysis of AF epidemiology, patient demographics, and treatment modalities in Korea, drawing from the extensive Korea National Health Insurance Service database.

## Highlights

• AF is the most common arrhythmia encountered in clinical practice, with its prevalence rising globally. However, a comprehensive update on AF epidemiology and treatment in Korea has been lacking for the past decade.
• From 2013 to 2022, AF prevalence doubled from 1.1% to 2.2%, while the incidence increased by 50% from 184 to 275 per 100,000 person-years.
• The average age of AF patients is rising, with a growing proportion aged 75 years or older (from 34.9% in 2013 to 43.9% in 2022), and CHA_2_DS_2_-VASc scores reflecting an increase in comorbidities.
• OAC use in patients with a CHA_2_DS_2_-VASc score ≥2 improved significantly from 44.6% to 77.5%, with DOACs becoming the dominant therapy.
• The use of AADs and AF ablation procedures has increased, although procedural rhythm control strategies remain underutilized.
• An integrated care approach is essential to enhance outcomes for AF patients, necessitating a comprehensive management strategy.

Atrial fibrillation (AF) is the most prevalent sustained cardiac arrhythmia, with its incidence increasing because of an aging population and associated comorbidities.^1^^,^^2^ The global burden of AF is rising steadily, driven by demographic shifts, particularly the aging of populations and the increasing prevalence of comorbidities such as hypertension, diabetes, and obesity. As a major contributor to cardiovascular disease, AF poses substantial challenges for both individual patient management and broader public health systems.^3^, ^4^, ^5^ AF is projected to affect 12 million individuals in the United States by 2050 and 17.9 million in Europe by 2060.^6^^,^^7^ In South Korea (hereafter, Korea), the epidemiology of AF has mirrored global trends, with a significant rise in incidence and prevalence over recent decades.^1^^,^^2^ The rapid aging of the Korean population, combined with a growing burden of lifestyle-related comorbidities, has contributed to a sharp increase in AF-related hospitalizations. Recent data indicate that AF-related hospital admissions have surged 4.2-fold over the past decade, while health care costs have escalated by approximately 5.7-fold.^5^

Patients with AF exhibit elevated risks of mortality and comorbidities, particularly heart failure (HF), dementia, and ischemic stroke, compared with individuals without AF.^8^, ^9^, ^10^ In recent years, there have been substantial advancements in both pharmacological and nonpharmacological therapies for AF. However, most of our current understanding of AF's incidence, prevalence, and associated cardiovascular morbidity and mortality stems from studies conducted in Western countries.

AF significantly contributes to ischemic stroke, a leading cause of morbidity and mortality worldwide. AF patients face a 5-fold higher stroke risk compared with those without AF, making stroke prevention crucial in AF management. Korea's rapidly aging population and the increasing prevalence of AF-related comorbidities are expected to substantially raise the AF burden, highlighting the need for the effective and widespread implementation of stroke prevention strategies to mitigate thromboembolic events. Recent years have seen a paradigm shift in managing AF-related stroke risk, with direct oral anticoagulants (DOACs) now at the forefront of stroke prevention. However, despite these advances, challenges persist in the widespread adoption of anticoagulation therapy, with gaps in treatment adherence and varying practice patterns across the country. Korean stroke prevention guidelines, aligned with international recommendations, advocate anticoagulant use in high-risk patients.^11^, ^12^, ^13^ However, nationwide statistics show that many eligible patients are undertreated or untreated.^14^^,^^15^ This underutilization of stroke prevention measures highlights the need for a more comprehensive understanding of current practices and barriers to optimal care.

In addition to stroke prevention, 2 primary strategies are utilized for AF rhythm control: pharmacological therapies, such as antiarrhythmic drugs (AADs); and nonpharmacological interventions, including catheter ablation. Although AADs are commonly prescribed, their long-term use can be challenging caused by varying efficacy, potential side effects, and the risk of proarrhythmic complications in some patients. Atrial fibrillation catheter ablation (AFCA) has become a highly effective nonpharmacological treatment for AF rhythm control, especially for patients unresponsive to or intolerant of AADs. Improved techniques like radiofrequency (RF) and cryoballoon ablation have led to better outcomes, with studies showing superior long-term rhythm control and quality of life compared with pharmacological treatments. In Korea, AFCA has increased steadily, becoming a cornerstone in AF rhythm control management.^16^ Despite progress, AF management in Korea faces challenges. Adoption of AADs and AFCA varies across regions caused by differences in medical technology access, health care infrastructure, and provider expertise. Underdiagnosis, late patient presentation, and inconsistent guideline adherence complicate effective AF management. Understanding nationwide trends in AF management—particularly the use of AADs and AFCA—is crucial for improving patient outcomes and shaping health care policy. Epidemiological data offer valuable insights into treatment patterns, intervention efficacy, and care disparities. These insights can guide efforts to enhance AF management across diverse patient populations.

Recently, the Korean Heart Rhythm Society reported an update on AF epidemiology, AF treatment, outcomes, and trends over the past decade in Korea based on a nationwide claims database.^17^, ^18^, ^19^ This paper aims to comprehensively review the temporal trends in the prevalence and incidence of AF in Korea, as well as the associated risks of cardiovascular events and all-cause mortality, using data from the National Health Insurance Service (NHIS) database, which encompasses the entire Korean population from 2013 to 2022. Additionally, it utilizes the most recent statistical data to provide a detailed analysis of AF management in Korea, with a particular focus on stroke prevention strategies. By evaluating trends in the use of anticoagulation therapy, treatment adherence, and patient outcomes, this study seeks to identify key areas for improvement and to inform future health care policies. A clearer understanding of current clinical practices will support efforts to mitigate the AF-related stroke burden and enhance patient outcomes nationwide. Finally, we will examine trends in AF management to assess the evolving utilization of AADs and catheter ablation, providing insights into the broader therapeutic landscape in Korea.

## Korean NHIS Data Source and Methodology

This report on AF epidemiology was based on an analysis of the Korean NHIS database. To briefly explain the formation and organization of this data source, the government of Korea provides the NHIS for all citizens, approximately 52 million people. It is mandatory for all Korean citizens (enrollees or beneficiaries) and provides universal and comprehensive insurance coverage for health care utilization.^20^^,^^21^ The NHIS makes payments based on billing records submitted by health care providers for medical expenditures. For this purpose, the NHIS has established an insurance eligibility database of enrollees, and expenditure information based on medical usage is collected for each enrollee so that diagnosis codes and medical usage details that led to medical usage are all systematically collected and managed.

The eligibility and health care utilization databases are mainly used for research purposes, and this AF epidemiology update report was analyzed based on the information in both databases. The eligibility database includes sociodemographic data, the use of inpatient and outpatient services, and data on death, as compiled by Statistics Korea. In contrast, the health care utilization database consists of inpatient and outpatient medical service use, including laboratory tests, prescription records, medical materials, procedures, surgery, and diagnostic codes. The diagnoses are coded through the Korean Standard Classification of Disease version 7, based on the International Classification of Disease-10th Revision (ICD-10). Data have been collected cumulatively since 2002 and are typically available up to the previous year at the time of a data request.

The NHIS database is accessible to researchers after fulfilling research criteria and receiving approval. The analysis is conducted on a designated PC, and although the raw data cannot be exported, the results of analyses can be. All data are anonymized to protect individual privacy, ensuring strict confidentiality of personal information.

For this analysis, we used data from the entire Korean population aged 20 years and older from January 1, 2002, to December 31, 2022. To analyze the prevalence and incidence of AF, we defined the index date as the first time ICD-10 codes I48.0-I48.4 and I48.9 were billed from January 1, 2002, to December 31, 2022.^1^, ^2^, ^3^, ^4^, ^5^ To accurately define AF, we used the existing literature to define a minimum of 2 claims for outpatient diagnoses (in which case the second claim is the index date) and a minimum of 1 claim for inpatient diagnoses.^1^, ^2^, ^3^, ^4^, ^5^ The definition of AF using diagnoses and medical utilization has been reported to have a positive predictive value of 94.1% in the previous study.^22^^,^^23^ Patients younger than 20 years of age and patients with valvular heart disease (defined as mitral stenosis [I05.0, I05.2, I05.9] or prosthetic heart valves [Z95.2-95.4]) were excluded from this analysis.

### Part I: Epidemiology of atrial fibrillation in Korea

This report will analyze the annual AF prevalence and incidence over the last 10 years, from 2013 to 2022.^17^ The annual prevalence of AF was determined by dividing the number of patients diagnosed with AF who were alive at the end of each year by the total population of Korean residents living at that time.^17^ The annual incidence of AF was the rate of newly diagnosed AF identified in health claims data within a given year.^17^ The year of new diagnosis was determined by the first recorded occurrence of AF-related ICD-10 codes, with the initial 11 years (2002 to 2012) excluded to prevent misclassification of preexisting AF as newly diagnosed. The annual incidence rate was calculated by dividing the number of new AF cases by the total person-years at risk among Korean residents without a prior AF diagnosis during that year. Incidence rates were reported per 100,000 person-years. Prevalence and incidence were stratified by sex (male and female) and age groups (20-29, 30-39, 40-49, 50-59, 60-69, 70-79, and ≥80 years). Comorbidities and adverse outcomes were analyzed to characterize the baseline profile of patients with prevalent AF between 2013 and 2022. Assessed comorbidities included hypertension, diabetes, HF, prior ischemic stroke, prior transient ischemic attack (TIA), myocardial infarction (MI), and peripheral artery disease, using data from up to 5 years preceding the year of AF prevalence. The CHA_2_DS_2_-VASc score was calculated based on these comorbidities, with detailed definitions provided in Supplemental Table 1.^23^ As patients age and develop more comorbidities, their CHA_2_DS_2_-VASc score—a measure of stroke risk—tends to rise. To explore this trend within a consistent population, a study was conducted on a cohort of 458,666 patients diagnosed with AF in 2017 who survived until 2022. This analysis tracked year-to-year changes in comorbidities and CHA_2_DS_2_-VASc scores over 5 years, providing insights into how stroke risk evolves over time in AF patients. Epidemiological trends of adverse outcomes, such as all-cause mortality, ischemic stroke, major bleeding, MI, HF hospitalization, and dementia, were also assessed. The diagnostic validity of these outcomes in the Korean NHIS database has been previously validated, with definitions detailed in Supplemental Table 1.^10^^,^^20^^,^^24^

The annual incidence rates of adverse outcomes (% per year) among patients with prevalent AF were calculated by dividing the number of first-time events each year by the total number of event-free patients at the start of that year. Adverse outcomes in the non-AF population were assessed using an age- and sex-matched cohort from the Korean NHIS-National Sample Cohort. Non-AF participants were selected via propensity score matching, using a 1:2 ratio with prevalent AF patients as the reference group at the start of 2017. The adverse event rates between the matched AF and non-AF cohorts were compared over a 3-year follow-up period (2017-2019) using Cox regression analysis.

### Part II: Stroke prevention in Korean patients with AF

Prescription rates of antithrombotic treatment in prevalent AF patients with CHA_2_DS_2_-VASc score ≥2 in men and ≥3 in women were analyzed from 2013 to 2022.^18^ Antithrombotic treatment includes antiplatelet agents and OACs. OACs include warfarin, apixaban, dabigatran, edoxaban, and rivaroxaban. The OAC prescription rate was calculated by considering the continued use of OACs in each period according to the prescription date and amount. Prescription data were obtained from NHIS outpatient encounter documentation. For patients with multiple encounters, the last encounter was entered into the analysis. Usage of OACs was defined as more than 30-day prescriptions within a 6-month AF diagnosis.

In Part II, we also reported the number and proportion of ischemic stroke events accompanied by newly diagnosed AF. It was defined as a case in which AF was diagnosed within 6 months before or after the ischemic stroke.

### Part III: Rhythm and rate management for Korean patients with AF: medication and catheter ablation

We examined the prescription trends of rate control drugs, AADs, and the utilization of AFCA for patients with prevalent and incident AF from 2013 to 2022.^19^ Rate control drugs included beta-blockers, nondihydropyridine calcium-channel blockers, and digoxin. AADs were categorized into class Ic and III agents. Class Ic included flecainide, propafenone, and pilsicainide, whereas class III included amiodarone, dronedarone, and sotalol. Patients who were prescribed AADs for more than 30 days within a given year were classified as AAD users for that specific year. This classification process was applied annually from 2013 to 2022. Prescription history was analyzed for all AADs, as well as separately for class Ic and III drugs. The total number of AFCA performed annually among patients with prevalent AF was assessed for each year from 2013 to 2022, including RF and cryoablation procedures. Pulsed-field ablation was not performed in Korea during the study period. Only de novo AFCA procedures were included. Cryoablation was introduced in June 2018. Early AFCA was defined as ablation conducted within 1 year of AF diagnosis. The total number of incident AF patients undergoing early AFCA was counted for each year from 2013 to 2021. Because follow-up data for 2023 were unavailable, the number of incident AF patients undergoing early AFCA for that year could not be assessed.

## Part I: Epidemiology of AF in Korea

### Temporal trends of prevalence and incidence of AF

**Figure 1:**
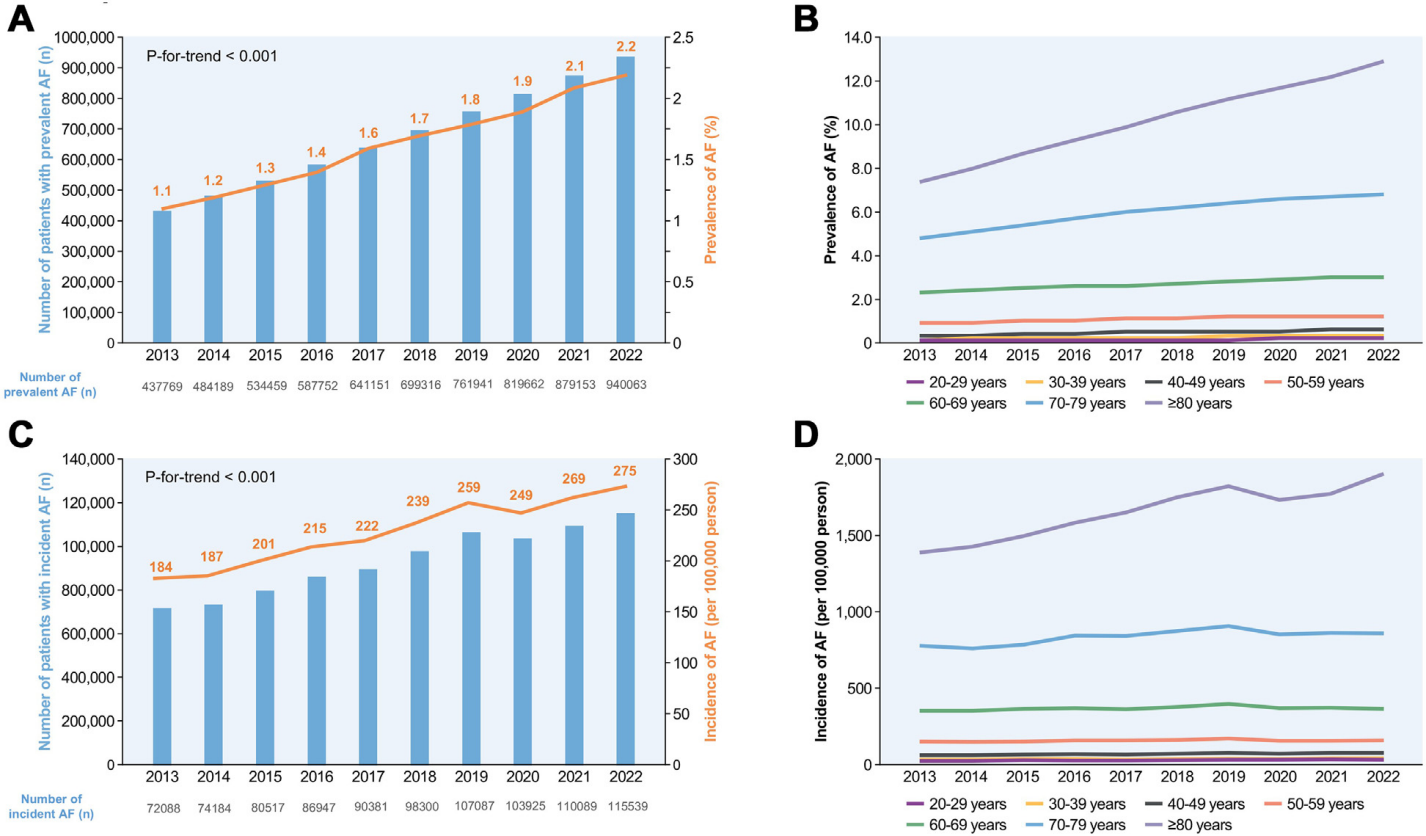
AF Prevalence and Incidence in Korea Between 2013-2022 (A) Prevalence of atrial fibrillation (AF). The overall prevalence of AF among Korean adults aged ≥20 years increased from 1.1% in 2013 to 2.2% in 2022. (B) Prevalence of AF by age groups. The rising trend was observed across all age groups, with a particularly steep increase in older adults. (C) Incidence of AF. The annual incidence of newly diagnosed AF increased from 184 to 275 per 100,000 person-years over the 10-year period. (D) Incidence of AF by age groups. This increase was most notable among those aged ≥80 years, with an incidence rate of 1,903 per 100,000 person-years in 2022. Adapted with permission from Lee et al.^17^

Temporal trends of prevalence and incidence of AF are described in Figure 1. Between 2013 and 2022, the prevalence of AF among Korean adults aged 20 years and older surged significantly. The number of AF patients increased from 43,769 in 2013 to 940,063 in 2022, with the overall prevalence doubling from 1.1% to 2.2% (*P* for trend <0.001) (Figure 1A). This upward trend was consistent across both men and women, with AF prevalence reaching 2.4% in men and 2.0% in women by 2022 (*P* for trend <0.001). The increase in AF prevalence was evident across all age strata, with a particularly pronounced effect among older adults. The annual increment in AF was more substantial in the elderly cohort, demonstrating a higher overall prevalence throughout the study period (Figure 1B, Supplemental Table 2). Regionally, there were variations in AF prevalence, with Jeonbuk having the highest rate at 3.5% and Sejong City having the lowest at 1.6%. The prevalence in suburban and rural areas (2.4%) was significantly higher than in urban regions (1.9%) (*P <* 0.001).

The incidence of newly diagnosed AF cases also showed a steady rise over the 10-year period. Between 2013 and 2022, the number of newly diagnosed AF patients each year steadily increased (Figure 1C). The incidence rose from 184 per 100,000 person-years in 2013 to 275 per 100,000 person-years in 2022, reflecting a 1.5-fold increase (*P* for trend <0.001) (Figure 1C). This upward trend in AF incidence was significant in both men and women, with the 2022 incidence rates reaching 301 per 100,000 person-years in men and 249 per 100,000 person-years in women. Similar to the prevalence trends, the incidence of AF was particularly high among older populations, with a sharp rise observed in individuals aged 80 years and above (Figure 1D, Supplemental Table 2). In 2022, the incidence rate for this age group reached 1,903 per 100,000 person-years, underscoring the strong correlation between age and AF incidence.

Although the aging population and rising comorbidities are primary drivers of the observed increase in AF prevalence and incidence, it is also plausible that enhanced awareness of AF among health care providers and the general population, coupled with improved diagnostic tools such as wearable electrocardiogram (ECG) devices, has contributed to earlier detection and reporting. In Korea, where regular health check-ups are widely practiced, asymptomatic AF cases are more easily detected, further influencing prevalence estimates. This increased awareness may partially account for the upward trends observed from 2013 to 2022, underscoring the need for nuanced interpretations of epidemiological data that consider both demographic shifts and diagnostic advancements.

In summary, the period from 2013 to 2022 witnessed a significant escalation in both AF prevalence and incidence in Korea, with a particularly pronounced effect among the elderly population. These findings underscore the growing public health concern posed by AF and highlight the need for targeted interventions and management strategies, especially for older adults.

### Changes in the characteristics of patients with AF

**Figure 2:**
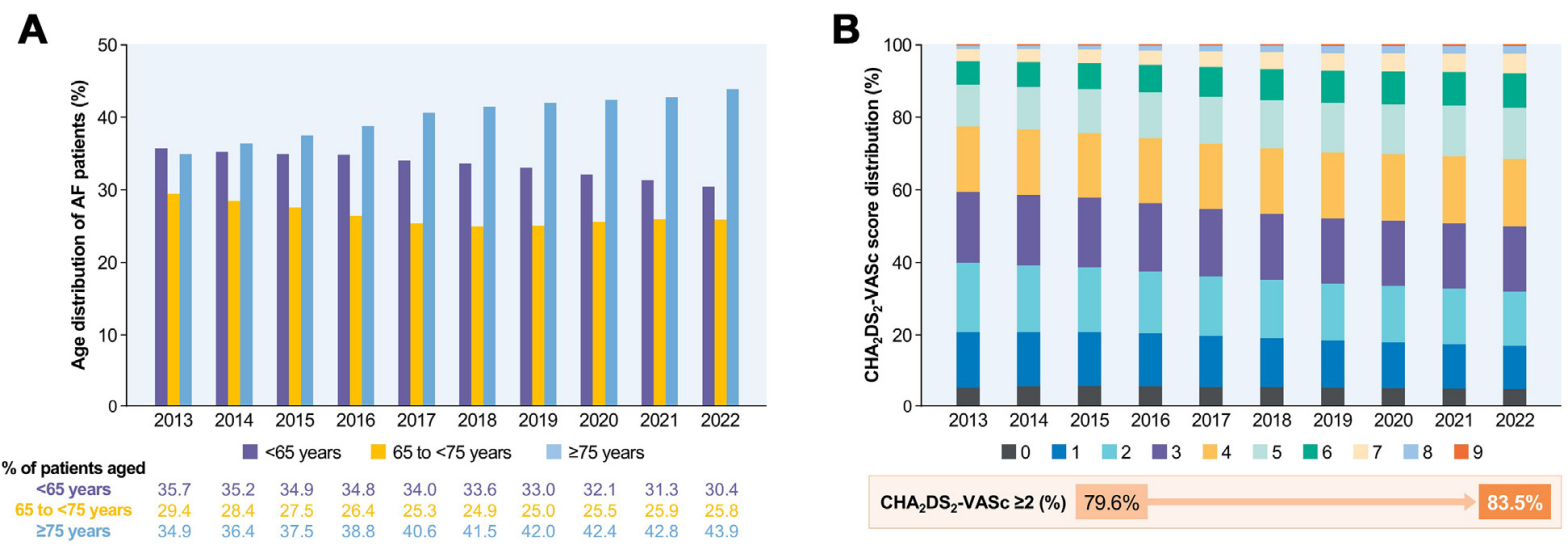
Age and CHA_2_DS_2_-VASc Score Distribution of Prevalent AF Patients Between 2013-2022 (A) Age distribution of prevalent atrial fibrillation (AF) patients between 2013 and 2022. The proportion of older adults aged ≥75 years among AF patients increased markedly from 34.9% in 2017 to 43.9% in 2022 (*P* for trend <0.001), reflecting the aging AF population. (B) CHA_2_DS_2_-VASc score distribution of prevalent AF patients between 2013 and 2022. The proportion of AF patients with a CHA_2_DS_2_-VASc score ≥2 increased from 79.6% in 2013 to 83.5% in 2022, indicating a growing trend toward a higher-risk AF population over time. Adapted with permission from Lee et al.^17^

Over time, AF patients demonstrate an increasing prevalence of high-risk characteristics, primarily driven by aging and a rise in associated comorbidities (Table 1). From 2013 to 2022, the average age of AF patients steadily increased from 67.7 to 70.3 years (*P* for trend <0.001). The proportion of older individuals aged ≥75 years increased significantly from 34.9% in 2017 to 43.9% in 2022 (*P* for trend <0.001) (Figure 2A). Concurrently, the prevalence of comorbidities such as hypertension, diabetes, HF, prior ischemic stroke, TIA, and MI also increased. This was accompanied by an elevation in the mean CHA_2_DS_2_-VASc score, from 3.2 ± 1.8 in 2013 to 3.6 ± 2 in 2022, along with a growing proportion of patients with higher CHA_2_DS_2_-VASc scores (*P* for trend <0.001) (Figure 2B).

**Table 1:** Characteristics of the Patients With Prevalent AF in Korea Between 2013 and 2022

	2013	2014	2015	2016	2017	2018	2019	2020	2021	2022	*P* for Trend
Prevalent AF, n	437,769	484,189	534,459	587,752	641,151	699,316	761,941	819,662	879,153	940,063	
Age (y), mean	67.7 ± 13.6	68 ± 13.7	68.2 ± 13.8	68.5 ± 13.9	68.8 ± 13.9	69.1 ± 14	69.4 ± 14	69.7 ± 14	70 ± 14.1	70.3 ± 14.1	<0.001
<65 y	156,166 (35.7)	170,433 (35.2)	186,737 (34.9)	204,488 (34.8)	218,190 (34.0)	234,820 (33.6)	251,421 (33.0)	262,768 (32.1)	275,372 (31.3)	285,694 (30.4)	<0.001
65 to <75 y	128,653 (29.4)	137,522 (28.4)	147,199 (27.5)	155,224 (26.4)	162,464 (25.3)	174,412 (24.9)	190,419 (25.0)	209,199 (25.5)	227,509 (25.9)	242,156 (25.8)	<0.001
≥75 y	152,950 (34.9)	176,234 (36.4)	200,523 (37.5)	228,040 (38.8)	260,497 (40.6)	290,084 (41.5)	320,101 (42.0)	347,695 (42.4)	376,272 (42.8)	412,213 (43.9)	<0.001
Men	237,632 (54.3)	263,218 (54.4)	290,750 (54.4)	319,823 (54.4)	349,381 (54.5)	381,861 (54.6)	417,009 (54.7)	449,253 (54.8)	482,553 (54.9)	516,477 (54.9)	<0.001
Hypertension	361,736 (82.6)	395,053 (81.6)	433,552 (81.1)	475,517 (80.9)	517,024 (80.6)	562,724 (80.5)	613,548 (80.5)	659,694 (80.5)	707,187 (80.4)	756,345 (80.5)	<0.001
Diabetes	118,050 (27.0)	131,536 (27.2)	145,773 (27.3)	162,068 (27.56)	179,464 (28.0)	198,619 (28.4)	220,901 (29.0)	243,078 (29.7)	267,726 (30.6)	295,798 (31.5)	<0.001
Heart failure	71,473 (16.3)	78,654 (16.2)	88,850 (16.6)	113,413 (19.3)	141,450 (22.1)	172,034 (24.6)	201,489 (26.4)	219,171 (26.7)	236,929 (27.0)	259,598 (27.6)	<0.001
Prior ischemic stroke	65,535 (15.0)	78,422 (16.2)	91,329 (17.1)	105,628 (18.0)	119,959 (18.7)	135,373 (19.4)	151,960 (19.9)	167,036 (20.4)	181,545 (20.7)	196,544 (20.9)	<0.001
Prior TIA	5,165 (1.2)	6,403 (1.3)	7,609 (1.4)	9,180 (1.6)	10,525 (1.6)	12,099 (1.7)	14,095 (1.9)	16,230 (2.0)	18,170 (2.1)	20,050 (2.1)	<0.001
Vascular disease	58,098 (13.3)	60,875 (12.6)	67,642 (12.7)	76,987 (13.1)	87,623 (13.7)	100,462 (14.4)	114,823 (15.1)	124,307 (15.2)	135,910 (15.5)	148,562 (15.8)	<0.001
Myocardial infarct	39,547 (9.0)	39,914 (8.2)	44,095 (8.3)	49,775 (8.5)	56,713 (8.9)	66,342 (9.5)	77,181 (10.1)	84,765 (10.3)	94,029 (10.7)	104,144 (11.1)	<0.001
PAD	21,068 (4.8)	23,533 (4.9)	26,474 (5.0)	30.688 (5.2)	3.5050 (5.5)	39,180 (5.6)	43,674 (5.7)	45,982 (5.6)	48,956 (5.6)	52,102 (5.5)	<0.001
CHA_2_DS_2_-VASc, mean	3.2 ± 1.8	3.2 ± 1.9	3.2 ± 1.9	3.3 ± 1.9	3.4 ± 1.9	3.4 ± 2	3.5 ± 2	3.5 ± 2	3.5 ± 2	3.6 ± 2	<0.001
≥2	348,580 (79.6)	384,844 (79.5)	425,082 (79.5)	470,287 (80.0)	517,531 (80.7)	568,568 (81.3)	614,556 (81.9)	676,076 (82.4)	729,217 (82.9)	785,150 (83.5)	<0.001

AF = atrial fibrillation; PAD = peripheral artery disease; TIA = transient ischemic attack.

As individuals age and accumulate additional comorbidities, their CHA_2_DS_2_-VASc score, reflecting stroke risk, tends to rise. An analysis of annual changes in comorbidities and CHA_2_DS_2_-VASc scores from 2017 to 2022 was conducted on a cohort of 458,666 patients diagnosed with AF in 2017 who survived through 2022. A notable increase was observed in the percentage of patients aged ≥75 years, climbing from 27.7% in 2017 to 42.3% in 2022 (*P* for trend <0.001). Among these individuals, comorbidities such as hypertension, diabetes, and HF are common, with hypertension being the most predominant. The prevalence of comorbidities such as hypertension, diabetes, HF, previous ischemic stroke, prior TIA, and MI grew over time. As a result, the mean CHA_2_DS_2_-VASc score increased from 2.9 ± 1.8 in 2017 to 3.4 ± 1.9 in 2022, and the proportion of patients with a score of ≥2 rose from 74.5% in 2017 to 81.7% in 2022.

Previous epidemiological research using the GARFIELD (Global Anticoagulant Registry in the FIELD) has found that around 70% of AF patients have hypertension, while roughly 20% have diabetes, aligning with the observations in Korea.^25^ Worldwide, AF accounts for 2.6% of the HF burden, with an estimated 1.5 million people living with both AF and HF in 2019, representing a 49.8% rise since 1990. The regions with the highest prevalence were South-East Asia, East Asia, and Oceania.^26^ The findings in the Korean population suggest that the rising prevalence of comorbidities with advancing age significantly impacts CHA_2_DS_2_-VASc score distribution, which in turn affects stroke risk. In light of these trends, optimizing anticoagulation approaches and improving comprehensive comorbidity management, such as the AF-CARE pathway and HEAD2TOES strategy, would be of paramount importance.^11^^,^^12^

### Risk of adverse clinical outcomes associated with AF

**Figure 3:**
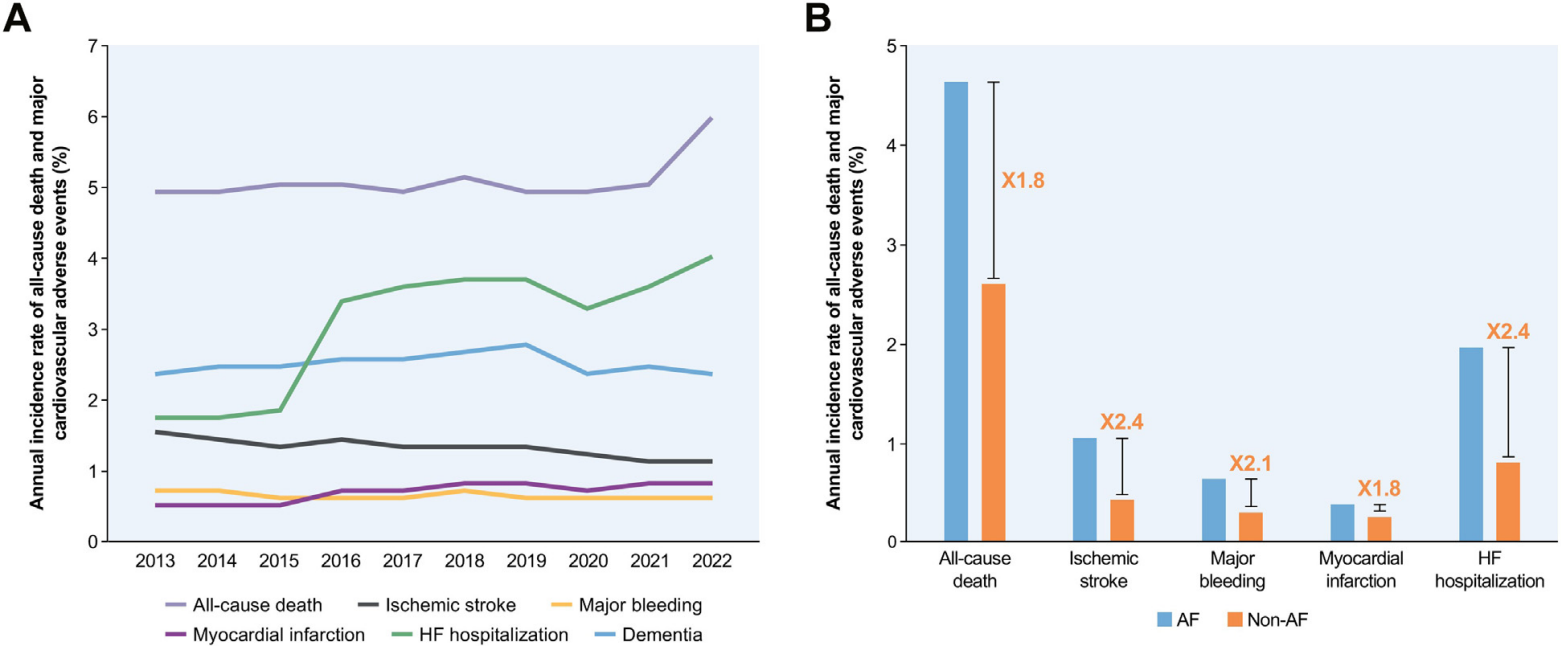
Clinical Outcome of Patients With AF (A) Annual incidence rates of clinical outcomes of atrial fibrillation (AF) patients between 2013 and 2022. From 2013 to 2022, the annual rates of ischemic stroke in AF patients declined, potentially reflecting increased use of oral anticoagulants. In contrast, rates of all-cause mortality, heart failure (HF) hospitalization, and myocardial infarction increased significantly (all *P* for trend <0.001), likely caused by aging and increased comorbidity burden. The rate of dementia remained stable. (B) Comparison between AF patients and age/sex-matched non-AF population of annual incidence rates of clinical outcomes. Compared with individuals without AF, patients with AF had significantly higher risks of adverse outcomes, including mortality (HR: 1.78), ischemic stroke (HR: 2.39), major bleeding (HR: 2.10), myocardial infarction (HR: 1.44), and HF hospitalization (HR: 2.42). Adapted with permission from Lee et al.^17^

An analysis of epidemiological trends in adverse outcomes among Korean patients AF revealed a decline in annual rates of ischemic stroke and major bleeding over the past decade (Figure 3A, Supplemental Table 3). This reduction may be attributed to the rapid increase in DOAC and overall OAC prescription rates following the implementation of full reimbursement for DOACs in 2015. However, the rates of all-cause mortality, HF hospitalization, and MI demonstrated an upward trend (all *P* for trend <0.001), emphasizing the growing prevalence of comorbidities with aging in the AF population in Korea (Figure 3A). The annual rate for dementia remained relatively constant.

The increase in HF hospitalization rates in recent years may reflect the aging AF cohort and rising comorbidity burden, such as hypertension and diabetes, heightening HF risk. The increased utilization of rhythm control treatments, including the use of AADs and AFCA from 2013 to 2022—which will be discussed later in this section—might be expected to reduce HF incidence. However, the utilization of rhythm control treatment still remains largely underutilized, suggesting room for improvement. Our reported HF incidence rates are crude, unadjusted for the progressive aging and higher comorbidity burden, including a rising prevalence of preexisting HF (16.3% in 2013 to 27.6% in 2022) (Table 1), which complicates interpretation. This limitation underscores the need for adjusted analyses to account for these factors. Although investigating this relationship is beyond the primary focus of our study, future research is warranted to explore the interplay between the penetration of rhythm control treatments, baseline patients’ age and comorbidity burden, and HF incidence, using detailed, adjusted analyses and patient-level data.

Although the direct impact of dementia on AF management is relatively limited compared with other cardiovascular complications, its clinical relevance is noteworthy. Previous studies have demonstrated that AF is associated with an increased risk of dementia, potentially medicated by cerebral hypoperfusion or thromboembolism, and that rhythm control strategies and appropriate OAC may mitigate this risk.^27^, ^28^, ^29^ The results showed a relatively constant annual dementia rate despite the aging and increasingly high-risk AF population, suggesting that improvement in the quality of AF management, including OAC treatment and rhythm control, may be attenuating progression. This observation underscores the critical need to prioritize dementia as a key outcome in future AF management, advocating for enhanced screening protocols and integrated care approaches, such as the AF-CARE pathway, to address both cardiovascular and cognitive health in AF patients.

Comparative analysis revealed that AF patients exhibited significantly elevated risks for various adverse outcomes relative to those without AF. These included increased risks of mortality (HR: 1.78; 95% CI: 1.67-1.89), ischemic stroke (HR: 2.39; 95% CI: 1.98-2.88), major bleeding (HR: 2.10; 95% CI: 1.53-2.87), MI (HR: 1.44; 95% CI: 1.09-1.91), and HF admission (HR: 2.42; 95% CI: 1.88-3.11) (Figure 3B). In comparison to meta-analysis findings largely based on non-Asian populations, Korean AF patients demonstrated higher relative risks for mortality (1.78 vs 1.46), comparable risks for ischemic stroke (2.39 vs 2.33), and lower risks for HF (2.42 vs 4.99), and MI (1.44 vs 1.61).^30^

Previous research from the Loire Valley AF project indicated that noncardiovascular causes accounted for 43% of deaths in AF patients, with HF, infection, and malignancy identified as the primary causes of mortality, rather than stroke-related events.^31^ Additionally, a separate study highlighted that HF, not stroke, was the most prevalent complication among AF patients. Approximately 2 in 5 patients developed HF, while 1 in 5 experienced a stroke during their remaining lifetime postdiagnosis, with minimal temporal improvement observed.^32^ These findings underscore the critical importance of optimizing both stroke prevention strategies and management of associated underlying diseases to enhance the overall care and outcomes for patients with AF.

## Part II: Stroke Prevention in Korean Patients With AF

### Temporal trends of antithrombotic therapy

**Figure 4:**
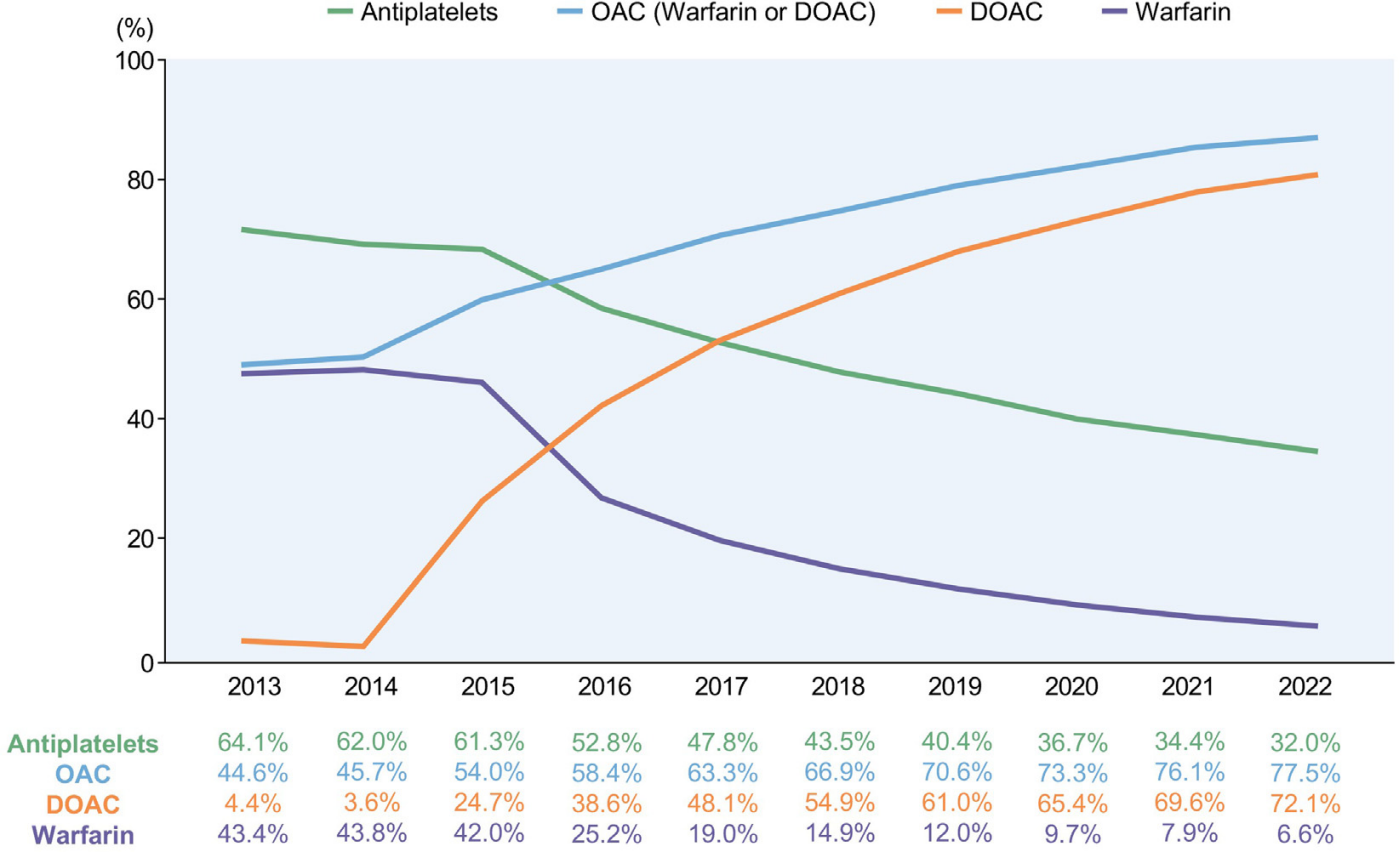
Prescription Rates of Antithrombotic Therapy in Prevalent AF Patients Between 2013-2022 Among prevalent atrial fibrillation (AF) patients with CHA2DS2-VASc score ≥2 in men and ≥3 in women, the proportion of those receiving oral anticoagulant (OAC) therapy increased from 44.6% in 2013 to 77.5% in 2022 (*P* < 0.001). Following full reimbursement of direct oral anticoagulants (DOACs) in 2015, DOAC use rose from 3.6% (2014) to 72.1% (2022), while warfarin use declined from 43.8% to 6.6% (*P <* 0.001 for both). Adapted with permission from Yang et al.^18^

The temporal trends in antithrombotic therapy for patients with AF in Korea demonstrated significant changes from 2013 to 2022. The proportion of patients receiving OAC treatment increased from 44.6% in 2013 to 77.5% in 2022 (*P <* 0.001) (Figure 4). Notably, there was no significant gender disparity in OAC prescription rates throughout this period. The introduction of full reimbursement for DOACs in 2015 precipitated a dramatic shift in prescribing patterns. DOAC prescription rates surged from 3.6% in 2014 to 24.7% in 2015, continuing to rise steadily to 72.1% by 2022 (*P <* 0.001). On the other hand, the prescription rate for warfarin dramatically decreased from 43.8% in 2014 to 6.6% in 2022 (*P <* 0.001). The individual prescription rate for each DOAC (apixaban, dabigatran, edoxaban, rivaroxaban) is expected to be unveiled after the patent expiration of all DOACs.

Concomitantly, antiplatelet therapy prescriptions decreased from 64.1% in 2013 to 32.0% in 2022 (*P <* 0.001). However, it is noteworthy that one-third of AF patients who are candidates for OAC therapy continue to receive antiplatelet agents, while more than 20% of AF patients do not receive any form of OAC. This observation underscores the need for enhanced education and interdisciplinary collaboration among physicians and interventionists to optimize antithrombotic therapy in AF patients, particularly those with recent percutaneous coronary intervention or concomitant non-embolic ischemic stroke.

Despite the introduction of full reimbursement for DOACs in the third quarter of 2015, the transition to widespread DOAC adoption in Korea took several years, reflecting a pattern observed globally.^33^, ^34^, ^35^, ^36^, ^37^ Initially, many physicians were cautious about prescribing DOACs, influenced by historical prescribing habits favoring antiplatelets, concerns about bleeding risks, and the lack of long-term real-world data specific to the Korean population. As domestic evidence on DOAC safety and effectiveness accumulated^38^, ^39^, ^40^—particularly alleviating fears of bleeding risks—DOAC prescription rates surged, increasing from 3.6% in 2014 to 72.1% in 2022, with overall OAC use improving from 44.6% in 2013 to 77.5% in 2022 in AF patients at high risk for stroke (CHA_2_DS_2_-VASc score ≥2 in men and ≥3 in women). Comparative data from international registries similar delays in OAC uptake following DOAC introduction.^33^, ^34^, ^35^, ^36^, ^37^ These global trends underscore that the initial lag in Korea is not unique but rather a common phenomenon, reflecting the time required for physician confidence, guideline adoption, and real-world evidence to evolve. This observation highlights the need for ongoing education, interdisciplinary collaboration, and policy initiatives to optimize antithrombotic therapy and address barriers to OAC use in AF patients.

Analysis of OAC prescription rates stratified by CHA_2_DS_2_-VASc scores in 2022 revealed a gradual increase from 26% to 80% as scores increased (Table 2). One-fourth of AF patients with low thromboembolic risk received OACs, whereas almost 20% of AF patients with high thromboembolic risk did not receive any OACs. When considering female gender as a stroke risk modifier rather than an independent risk factor, no significant differences in OAC prescription rates were observed between men and women at each CHA_2_DS_2_-VASc score level.

**Table 2:** Oral Anticoagulant Prescription Rate in Atrial Fibrillation Patients According to CHA_2_DS_2_-VASc Score in 2022

CHA_2_DS_2_-VASc Score	Total (%)	CHA_2_DS_2_-VA Score	Total (%)	Men (%)	Women (%)
0	25.8	0	26.1	25.8	26.5
1	41.8	1	47.4	44.5	54.1
2	66.7	2	70.6	70.2	71.5
3	76.4	3	77.8	78.5	76.7
4	78.6	4	79.3	80.3	78.2
5	79.6	5	80.3	81.5	79.0
6	80.0	6	80.5	81.7	79.4
7	79.7	7	80.0	80.5	79.6
8	79.6	8	78.0	79.2	77.0
9	77.0	—		—	—

These findings highlight the evolving landscape of antithrombotic therapy in AF management in Korea and underscore the need for continued efforts to optimize treatment strategies in alignment with current guidelines and patient-specific risk factors.

### Strokes with AF and OAC prescription rates post-AF diagnosis

From 2013 to 2022, 20.4% of ischemic strokes were accompanied by AF in Korea, which was similar to previous reports.^41^ The incidence of patients receiving their initial AF diagnosis poststroke exhibited a steady increase, rising from 4,893 in 2013 to 6,978 in 2022. Over the decade spanning 2013 to 2022, 58,617 patients were first diagnosed with AF after experiencing a stroke. The proportion of such patients among first-diagnosed AF patients each year also increased from 5.5% in 2013 to 6.0% in 2022. This observation, wherein 5% to 6% of newly diagnosed AF cases were identified only after a stroke occurrence, underscores the imperative for enhanced AF screening protocols in high-risk populations. the implementation of more rigorous ECG screening measures, followed by appropriate OAC therapy, could potentially mitigate a significant proportion of these stroke events.

In Korea, a substantial number of patients diagnosed with AF do not receive appropriate OAC prescriptions. Among patients diagnosed with AF for the first time, 85% were at high risk for stroke (CHA_2_DS_2_-VASc score ≥2 in men and ≥3 in women) and required OAC prescriptions. However, 51% of these patients were not prescribed OAC even after 6 months of being diagnosed with AF. Despite an overall increase in OAC prescription rates over time, 4% of patients who did not receive OAC within 6 months of AF diagnosis subsequently experienced a stroke without ever being prescribed OAC therapy. The number of patients already diagnosed with AF but not prescribed appropriate OACs until a stroke occurred was 22,987 over 10 years (2013-2022).

These findings highlight the significant burden of ischemic strokes in Korea potentially attributable to undiagnosed or undertreated AF. The data suggest that enhancing AF screening protocols and ensuring timely initiation of OAC therapy could substantially reduce the incidence of AF-related strokes, emphasizing the critical need for improved strategies in AF management and stroke prevention.

### Regional differences in OAC prescription rates

Regional variations in OAC prescription rates for AF patients at high risk of stroke (CHA_2_DS_2_-VASc score ≥2 in men and ≥3 in women) were observed across Korea. The OAC prescription rates ranged from a nadir of 64.9% in Jeollabuk-do to a zenith of 82.1% in Jeju-do. Notably, suburban and rural regions exhibited a lower rate of OAC prescriptions compared with urban areas (76.0% vs 79.6%; *P* < 0.001). These findings corroborate previous research that identified similar geographical disparities in OAC utilization.^42^

The observed regional differences in OAC prescription rates may be attributed to multiple factors. Health care accessibility emerges as a primary concern, with rural areas experiencing a dearth of specialist care, particularly cardiologists, potentially leading to delayed AF diagnosis and suboptimal OAC prescribing practices. Furthermore, rural populations tend to be older and may have lower disease awareness, potentially resulting in a diminished understanding of the critical role of anticoagulation therapy.

Socioeconomic factors may also contribute to these disparities. Previous studies have demonstrated a positive correlation between income levels and OAC prescription rates, with higher-income groups more likely to DOACs.^42^ This socioeconomic gradient in OAC utilization persists despite the implementation of full reimbursement policies for DOACs.^43^

To address these regional and socioeconomic inequalities in OAC prescribing patterns, a multifaceted approach is warranted. This should encompass enhanced communication and education initiatives regarding AF and its management, coupled with efforts to ensure equitable access to health care services and medications across all geographical regions. Such strategies may help mitigate the current disparities and improve overall stroke prevention in AF patients throughout Korea.

## Part III: Rhythm and Rate Management for Korean Patients With AF: Medication and Catheter Ablation

### Trends in AF treatment: rate and rhythm control medication

The treatment of AF in has undergone substantial evolution in the utilization of rate and rhythm control medications over the past decade. As highlighted in the Korea AF Fact Sheet 2024,^19^ the prescription of AADs and rate control agents has exhibited a consistent upward trajectory, reflecting an alignment with international guidelines for optimal AF pharmacotherapy.

Regarding rate control, beta-blockers have emerged as the predominant therapeutic option, with a steady increase in utilization. In 2022, 46.6% of patients with AF were treated with beta-blockers, making them the predominant rate control medication. This trend aligns with global recommendations advocating beta-blockers as first-line therapy.^11^ In contrast, the use of calcium-channel blockers and digoxin has steadily declined. The diminishing prescription of nondihydropyridine calcium-channel blockers, such as verapamil and diltiazem, can be attributed to concerns regarding their potential adverse effects in patients with HF with reduced ejection fraction (left ventricular ejection fraction ≤40%), where these agents are contraindicated according to AF guidelines. Digoxin, once a mainstay of rate control, has experienced a dramatic reduction in prescriptions, primarily caused by studies indicating an elevated mortality risk, particularly when used as monotherapy in AF patients.^44^^,^^45^

**Figure 5:**
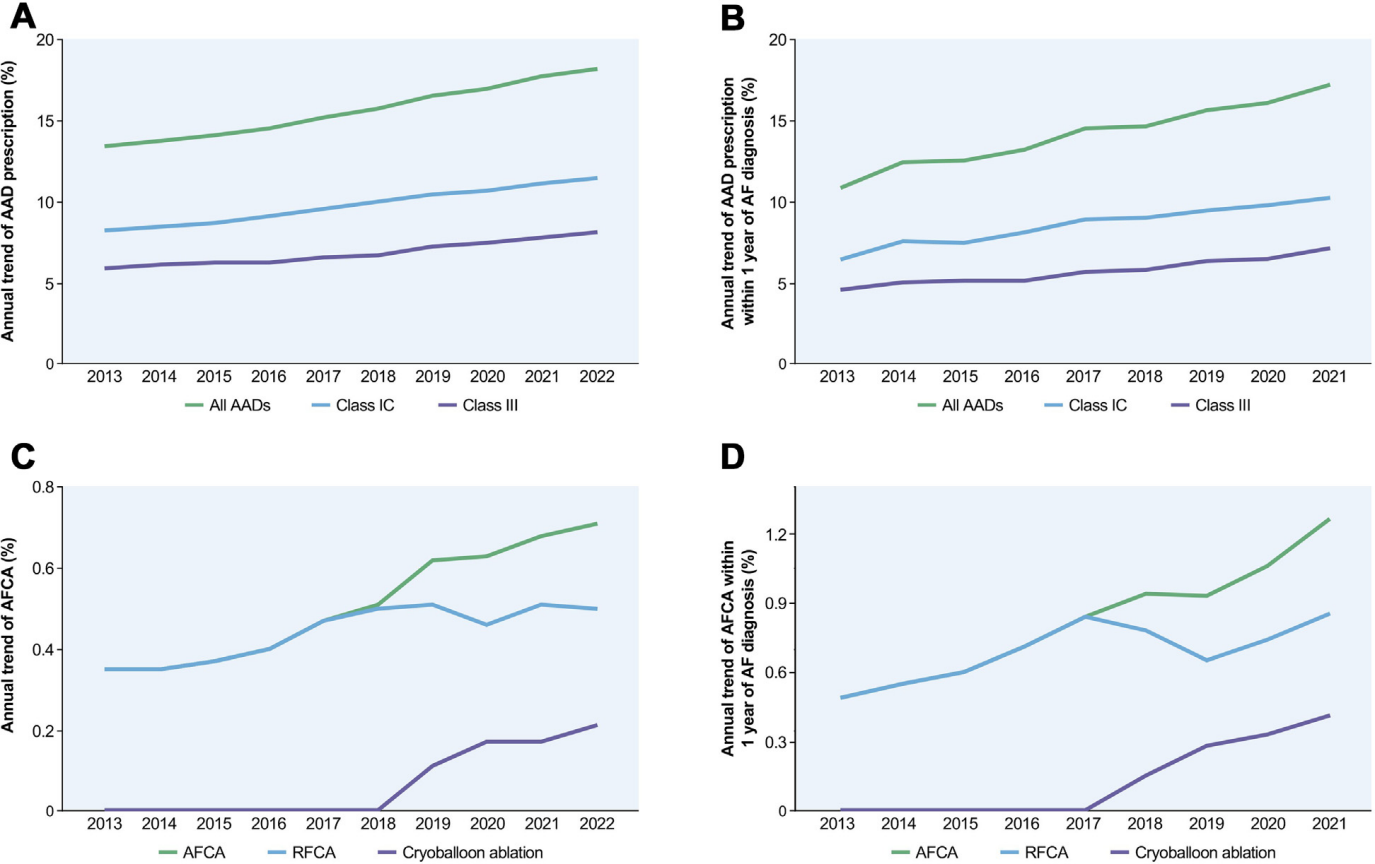
Utilization of AF Rhythm Control Treatment Between 2013-2022 (A, B) The use of antiarrhythmic drugs (AADs) gradually increased from 2013 to 2022, both in the overall atrial fibrillation (AF) population (A) and in newly diagnosed cases (B). Annual trends of atrial fibrillation catheter ablation (AFCA) (C) in patients with prevalent AF and (D) within 1 year after AF diagnosis. (C, D) The rate of AFCA steadily rose over the study period, particularly after the introduction of cryoablation in 2018. Although radiofrequency catheter ablation (RFCA) remained the dominant modality, cryoablation adoption grew rapidly. Early AFCA—performed within 1 year of diagnosis—also increased from 0.49% in 2013 to 1.26% in 2021, reflecting a shift toward earlier rhythm control in Korean clinical practice. In panels C and D, the AFCA curve represents the combined total of RFCA and cryoballoon ablation procedures. Adapted with permission from Kim et al.^19^

Rhythm control medications, which aim to restore and maintain normal sinus rhythm, have also seen significant changes (Figure 5A). The use of AADs for rhythm control has increased gradually, with 16.4% of AF patients receiving AADs in 2022, compared with 12.1% in 2013. This upward trend reflects an increasing emphasis on rhythm control, particularly in early-stage AF, aimed at mitigating progression to more persistent forms. This approach is supported by emerging evidence suggesting that early rhythm control may confer long-term benefits in AF patients.^46^ Class Ic drugs, such as flecainide and propafenone, are the most frequently prescribed AADs for patients with prevalent AF. A similar trend was observed among newly diagnosed AF patients (Figure 5B). These agents are generally preferred in patients without significant structural heart disease caused by their relatively favorable safety profile. In contrast, class III drugs, including amiodarone, are less commonly utilized, primarily caused by concerns regarding long-term toxicity risks. These changes in AAD use reflect advances in AF management, aligning with international guidelines that emphasize safety and an individualized approach. As the prevalence of AF continues to rise, optimizing the use of these medications will remain a fundamental focus to improve the outcomes of AF patients in Korea.

### Trend in AF treatment: catheter ablation

The number of de novo AFCA cases performed every year between 2013 and 2022 among prevalent AF patients was analyzed (Figure 5C). This analysis encompassed both RF and cryoablation procedures, with no pulsed-field ablation performed during the study period. Additionally, the trend for early AFCA, defined as procedures performed within 1 year of AF diagnosis, was evaluated.

In Korea, both the number and percentile value of AF patients undergoing AFCA gradually increased. In 2013, 1,528 patients (0.35% among prevalent AF patients) underwent AFCA, which increased to 6,652 (0.71%) in 2022. The rapid increase in AFCA procedures is noteworthy in addition to the increase in the number of prevalent AF patients, which is reflected by a 4-fold increase in absolute AFCA case number but a 2-fold increase in the percentage of patients undergoing AFCA. After introducing cryoablation in Korea in 2018, the number of patients increased dramatically from 23 to 1990 in 2022. In 2022, RF ablation was performed twice more frequently than cryoablation (4,682 vs 1,990 patients). The number of AFCA procedures performed in Korea plateaued during 2019 and 2020, probably because of the COVID-19 pandemic, but has since resumed its upward trajectory in the postpandemic period. With advancements in AFCA technologies, including sophisticated 3-dimensional mapping systems, novel energy delivery strategies such as high-power short-duration ablation, and emerging energy sources like cryoablation and pulsed field ablation, a substantial increase in AFCA utilization is anticipated in Korea. Although the number of patients receiving AFCA is increasing rapidly and is anticipated to increase further, still the majority of AF patients in Korea do not undergo AFCA, with only 0.71% among prevalent AF patients having AFCA in 2022. Due to the aging of society and the widespread of wearable ECG devices such as patch- or watch-ECG, the prevalence of AF is estimated to increase rapidly. Therefore, improving the quality of AFCA and increasing the number of AFCA procedures will be important tasks for electrophysiologists in Korea.

The importance of early treatment of AF has been emphasized recently, especially after the publication of the EAST-AFNET4 (Early treatment of Atrial Fibrillation for Stroke prevention Trial) trial, which demonstrated a 21% reduction in the composite outcome of death from cardiovascular causes, stroke, or hospitalization with worsening of HF or acute coronary syndrome.^46^ In the EARLY-AF (Early Aggressive Invasive Intervention for Atrial Fibrillation) trial, initial treatment with cryoablation rather than AAD therapy in paroxysmal AF patients was associated with a lower incidence of progression to persistent AF.^47^ Although a small portion of patients (0.49%) received early AFCA among those diagnosed with incident AF in 2013 in Korea, the number of early AFCA increased to up to 1.26% in 2021 (Figure 5D). Several potential reasons can exist for such a low performance rate of early AFCA in Korea. First, there are fewer electrophysiologists and dedicated electrophysiology labs than in the United States or Japan. Second, the current reimbursement policy only allows the performance of AFCA after 6 weeks of mandatory AAD treatment. Recent AF guidelines published by American College of Cardiology/American Heart Association/American College of Clinical Pharmacy/Heart Rhythm Society and European Society of Cardiology recommend AFCA to be performed as first-line therapy for both selected patients (Class 1 recommendation) and the others (Class 2a recommendation).^11^^,^^12^ Considering the aging population and the projected rapid increase in AF prevalence and incidence, the medical need for AFCA is expected to grow concurrently.^1^^,^^2^^,^^17^ Therefore, the medical need for AFCA will also grow in conjunction, and the effort to catch-up with the demand will be an important task for electrophysiologists in Korea. Detailed information on Figure 5 is described in Supplemental Table 4.

### Regional variations in rhythm control treatment

In Korea, catheter ablation for AF is predominantly performed in urban tertiary referral centers, where the majority of dedicated electrophysiologists are concentrated. This centralization of specialized care creates disparities in access to rhythm control therapies, including AFCA and AADs, for individuals residing in rural areas distant from these tertiary medical facilities. Indeed, the prescription rates of AADs and the performance rate of AFCA differed among regions in Korea. The prescription rate of AADs in 2022 was highest among people who lived in Seoul (19.2%) and Sejong (19.7%). The performance rate of AFCA was also highest among individuals who reside in Seoul (1.2%) and Sejong city (1.4%). The lowest AAD prescription and AFCA performance rate was observed in people living in Gyeongsangnam-do. In general, rural areas showed a significantly lower rate of AAD prescription and AFCA performance rate in Korea. As mentioned in Part II, OAC use in suburban and rural areas was lower compared with urban regions despite more severe comorbidities, mainly caused by limited medical access, lower AF management awareness, and differences in income and education level.^42^ Differences in clinical outcomes of AF can exist according to region. A recent study conducted with data within the province of Nova Scotia, Canada, demonstrated that AF patients living in rural locations had higher rates of recurrent AF-related emergency department visits and unplanned cardiovascular hospitalizations.^48^ Higher rates of re-visits or unplanned admissions in rural areas compared to urban areas might originate from limited accessibility to more advanced AF treatments such as AADs prescribed by electrophysiologists or the performance of AFCA.

To address these disparities, efforts should focus on improving access to tertiary medical centers and electrophysiologists in rural areas. Enhanced education for both patients and primary care physicians regarding the importance of adequate anticoagulation and rhythm control is crucial. Additionally, the establishment of a robust referral system could facilitate more equitable access to specialized AF care across diverse geographical regions.

### Opportunities for enhancing rhythm control and policy in Korean AF management

Although patient characteristics and guideline-directed management of AF share similarities globally, our investigation reveals specific opportunities for improvement in Korea, particularly in rhythm control strategies. Although OAC penetration has improved significantly—rising from 44.6% in 2013 to 77.5% in 2022—the utilization of rhythm control, such as AADs and AFCA, remains suboptimal, with only 0.71% of prevalent AF patients undergoing AFCA in 2022. These findings suggest that a more proactive and earlier approach to rhythm control could be beneficial, supported by emerging evidence from trials like EAST-AFNET4 and EARLY-AF, which demonstrate reduced progression to persistent AF and improved outcomes with early intervention.^46^^,^^47^ Additionally, advancements in AFCA technologies—such as reduced procedural complications, shorter procedure times, and simpler techniques—support the potential for increased adoption. This study has already informed the Korea AF Factsheet 2024, shaping national discussions on AF management, and we propose that these insights could guide updates to Korean AF guidelines and health care policies, advocating for greater emphasis on early rhythm control and addressing regional disparities in access to specialized care to optimize patient outcomes.

## Conclusions

Analysis of the Korean nationwide health care claims database reveals a significant increase in AF prevalence and incidence over the past decade (Central Illustration). The introduction of DOACs has markedly influenced anticoagulation prescription patterns for stroke prevention, leading to improved outcomes despite historically low anticoagulation rates in Asian populations. Although advancements have been made in rhythm and rate management, a substantial proportion of patients remain undertreated. These comprehensive epidemiological data from Korea underscore the need for comparative studies on AF management and outcomes across Asian countries. Such research would enhance our understanding of regional variations and facilitate the refinement of AF care strategies tailored to the Asian population, ultimately improving patient outcomes and reducing the burden of AF-related complications.

**Central Illustration:**
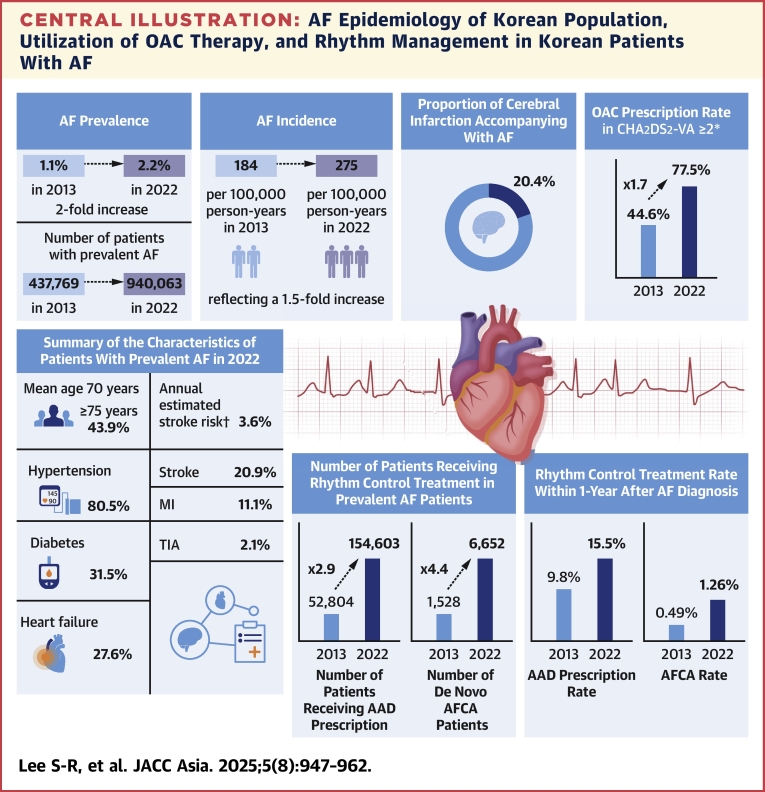
AF Epidemiology of Korean Population, Utilization of OAC Therapy, and Rhythm Management in Korean Patients With AF In Korea, the prevalence of atrial fibrillation (AF) has doubled, and the incidence of AF has increased by 1.5 times from 2013 to 2022, and AF patients are gradually becoming an aging and high-risk group. After the introduction of direct oral anticoagulants (OACs), the OAC rate increased by about 2 times, and the prescription of antiarrhythmic drug (AAD) for rhythm control and the implementation of AF catheter ablation are increasing significantly. AFCA = atrial fibrillation catheter ablation; MI = myocardial infarction; TIA = transient ischemic attack. ∗Indicates CHA_2_DS_2_-VASc score ≥2 in men and ≥3 in women. †Indicates mean CHA_2_DS_2_-VASc score.

## Funding Support and Author Disclosures

Dr Choi has received research grants or speaker fees from Abbott, Bayer, Bristol Myers Squibb/Pfizer, Biosense Webster, Chong Kun Dang, Daewoong Pharmaceutical Co, Daiichi-Sankyo, DeepQure, Dreamtech Co, Ltd, Jeil Pharmaceutical Co Ltd, Medtronic, Samjinpharm, Samsung Electronics Co, Ltd, Seers Technology, and Skylabs. All other authors have reported that they have no relationships relevant to the contents of this paper to disclose.
