# Supplementary material for: Nationwide Epidemiology and Management Time Trends for Atrial Fibrillation: Insights From the Korean AF Factsheet
Source: JACC Asia. 2025 May 27;5(8):947–62. doi: 10.1016/j.jacasi.2025.03.012 (PMC12426846; doi:10.1016/j.jacasi.2025.03.012)
Supplement: Supplemental Tables 1-4 [file mmc1.docx]

**Supplemental Tables**

**Supplemental Table 1. Definition of comorbidities and outcomes**

**Supplemental Table 2. AF prevalence and incidence between 2013 and 2022 by age groups**

**Supplemental Table 3. Annual incidence rates of all-cause death and major cardiovascular adverse events of AF patients**

**Supplemental Table 4. Annual trends of AAD prescription and AFCA between 2013 and 2022**

**Supplemental Table 1. Definition of comorbidities and outcomes**

| **Diagnosis** | **ICD-10 code and other operational conditions for definition** | **Diagnostic definition** |
| --- | --- | --- |
| **Comorbidities** |  |  |
| **Hypertension** | I10-I13, I15; and minimum 1 prescription of anti-hypertensive drug (thiazide, loop diuretics, aldosterone antagonist, alpha-/beta-blocker, calcium-channel blocker, angiotensin-converting enzyme inhibitor, angiotensin II receptor blocker). | Admission ≥1 or outpatient department ≥2 |
| **Diabetes mellitus** | E11-E14; and minimum 1 prescription of anti-diabetic drugs (sulfonylureas, metformin, meglitinides, thiazolidinediones, dipeptidyl peptidase-4 inhibitors, α-glucosidase inhibitors and insulin). | Admission ≥1 or outpatient department ≥1 |
| **Heart failure** | I50 | Admission ≥1 |
| **Prior ischemic stroke** | I63, I64 | Admission ≥1 |
| **Prior TIA** | G458, G459 | Admission or outpatient department ≥1 |
| **Vascular disease** | Composite of prior myocardial infarction and peripheral artery disease |  |
| **Myocardial infarction** | I21, I22 | Admission or outpatient department ≥1 |
| **Peripheral artery disease** | I70, I73 | Principal diagnosis; Admission or outpatient department ≥1 |
| **Clinical outcomes** |  |  |
| **Ischemic stroke** | I63-I64; with concomitant imaging studies (CT or MRI) of the brain or related death | Admission ≥1 |
| **Major bleeding** | Composite of intracranial hemorrhage and gastrointestinal bleeding |  |
| **Intracranial hemorrhage** | I60-62 with concomitant imaging studies (CT or MRI) of the brain or related death | Admission≥1 or RBC transfusion≥1 |
| **Gastrointestinal bleeding** | I85, K22.1, I22.8, K25.0, K25.2, K25.4, K25.6, K26.0, K26.2, K26.4, K26.6, K27.0, K27.2, K27.4, K27.6, K28.0, K28.2, K28.4, K28.6, K29.01, K29.21, K29.31, K29.41, K29.51, K29.61, K29.71, K29.81, K29.91, K31.8, K92.0, K92.1, K92.2, K55.2, K57.0, K57.1, K57.2, K57.3, K57.4, K57.5, K57.8, K57.9, K62.5, K66.1 | Admission≥1 and RBC transfusion≥1 |
| **Myocardial infarction** | I21-I22 | Admission ≥1 |
| **Heart failure admission** | I50 | Admission ≥1 |

Abbreviations: CT, computed tomography; MRI, magnetic resonance imaging; TIA, transient ischemic attack.

**Supplemental Table 2. AF prevalence and incidence between 2013 and 2022 by age groups**

|  | **2013** | | **2014** | **2015** | **2016** | **2017** | **2018** | **2019** | **2020** | **2021** | **2022** |  |
| --- | --- | --- | --- | --- | --- | --- | --- | --- | --- | --- | --- | --- |
| **Prevalence (%)** | | | | | | | | | | | | |
| **Age group, years** | |  |  |  |  |  |  |  |  |  |  |  |
| **20-29** | | 0.1 | 0.1 | 0.1 | 0.1 | 0.1 | 0.1 | 0.1 | 0.2 | 0.2 | 0.2 |  |
| **30-39** | | 0.1 | 0.2 | 0.2 | 0.2 | 0.2 | 0.2 | 0.3 | 0.3 | 0.3 | 0.3 |  |
| **40-49** | | 0.3 | 0.3 | 0.4 | 0.4 | 0.4 | 0.5 | 0.5 | 0.5 | 0.6 | 0.6 |  |
| **50-59** | | 0.9 | 0.9 | 1.0 | 1.0 | 1.1 | 1.1 | 1.2 | 1.2 | 1.2 | 1.2 |  |
| **60-69** | | 2.3 | 2.4 | 2.5 | 2.6 | 2.6 | 2.7 | 2.8 | 2.9 | 3.0 | 3.0 |  |
| **70-79** | | 4.8 | 5.1 | 5.4 | 5.7 | 6.0 | 6.2 | 6.4 | 6.6 | 6.7 | 6.8 |  |
| **≥ 80** | | 7.4 | 8.0 | 8.7 | 9.3 | 9.9 | 10.6 | 11.2 | 11.7 | 12.1 | 12.9 |  |
| **Incidence (per 100,000 person-years)** | | | | | | | | | | | | |
| **Age group, years** | |  |  |  |  |  |  |  |  |  |  |  |
| **20-29** | | 20 | 20 | 25 | 23 | 23 | 26 | 29 | 28 | 32 | 29 |  |
| **30-39** | | 29 | 28 | 32 | 32 | 29 | 34 | 36 | 34 | 38 | 38 |  |
| **40-49** | | 58 | 58 | 64 | 65 | 64 | 68 | 73 | 67 | 73 | 72 |  |
| **50-59** | | 148 | 146 | 149 | 155 | 155 | 159 | 168 | 152 | 154 | 155 |  |
| **60-69** | | 356 | 350 | 363 | 368 | 361 | 375 | 395 | 367 | 370 | 364 |  |
| **70-79** | | 777 | 760 | 786 | 844 | 842 | 874 | 908 | 853 | 863 | 860 |  |
| **≥ 80** | | 1392 | 1428 | 1500 | 1587 | 1653 | 1755 | 1827 | 1737 | 1775 | 1903 |  |

Abbreviation: AF, atrial fibrillation

**Supplemental Table 3. Annual incidence rates of all-cause death and major cardiovascular adverse events of AF patients**

|  | **2013** | **2014** | **2015** | **2016** | **2017** | **2018** | **2019** | **2020** | **2021** | **2022** |
| --- | --- | --- | --- | --- | --- | --- | --- | --- | --- | --- |
| **All-cause death** | 4.9% | 4.9% | 5.0% | 5.0% | 4.9% | 5.1% | 4.9% | 4.9% | 5.0% | 5.9% |
| **Ischemic stroke** | 1.6% | 1.5% | 1.4% | 1.5% | 1.4% | 1.4% | 1.4% | 1.3% | 1.2% | 1.2% |
| **Major bleeding** | 0.8% | 0.8% | 0.7% | 0.7% | 0.7% | 0.8% | 0.7% | 0.7% | 0.7% | 0.7% |
| **MI** | 0.6% | 0.6% | 0.6% | 0.8% | 0.8% | 0.9% | 0.9% | 0.8% | 0.9% | 0.9% |
| **HF hospitalization** | 1.8% | 1.8% | 1.9% | 3.4% | 3.6% | 3.7% | 3.7% | 3.3% | 3.6% | 4.0% |
| **Dementia** | 2.4% | 2.5% | 2.5% | 2.6% | 2.6% | 2.7% | 2.8% | 2.4% | 2.5% | 2.4% |

Abbreviation: AF, atrial fibrillation; HF, heart failure; MI, myocardial infarction.

**Supplemental Table 4. Annual trends of AAD prescription and AFCA between 2013 and 2022**

|  | **2013** | **2014** | **2015** | **2016** | **2017** | **2018** | **2019** | **2020** | **2021** | **2022** |
| --- | --- | --- | --- | --- | --- | --- | --- | --- | --- | --- |
| **Trend of annual prescription of AAD (%)** | | | | | | | | | | |
| **All AADs** | 12.1 | 12.4 | 12.7 | 13.1 | 13.7 | 14.2 | 14.9 | 15.3 | 16.0 | 16.4 |
| **Class Ic** | 7.4 | 7.6 | 7.8 | 8.2 | 8.6 | 9.0 | 9.4 | 9.6 | 10.0 | 10.3 |
| **Class III** | 5.3 | 5.5 | 5.6 | 5.6 | 5.9 | 6.0 | 6.5 | 6.7 | 7.0 | 7.3 |
| **Trend of annual prescription of AAD within 1-year of AF diagnosis (%)** | | | | | | | | | | |
| **All AADs** | 9.8 | 11.2 | 11.3 | 11.9 | 13.1 | 13.2 | 14.1 | 14.5 | 15.5 | - |
| **Class Ic** | 5.8 | 6.8 | 6.7 | 7.3 | 8.0 | 8.1 | 8.5 | 8.8 | 9.2 | - |
| **Class III** | 4.1 | 4.5 | 4.6 | 4.6 | 5.1 | 5.2 | 5.7 | 5.8 | 6.4 | - |
| **Trend of annual AFCA (%)** | | | | | | | | | | |
| **AFCA** | 0.35 | 0.35 | 0.37 | 0.40 | 0.47 | 0.51 | 0.62 | 0.63 | 0.68 | 0.71 |
| **RFCA** | 0.35 | 0.35 | 0.37 | 0.40 | 0.47 | 0.50 | 0.51 | 0.46 | 0.51 | 0.50 |
| **CBA** | 0.0 | 0.0 | 0.0 | 0.0 | 0.0 | 0.0 | 0.11 | 0.17 | 0.17 | 0.21 |
| **Trend of annual AFCA within 1-year of AF diagnosis (%)** | | | | | | | | | | |
| **AFCA** | 0.49 | 0.55 | 0.60 | 0.71 | 0.84 | 0.94 | 0.93 | 1.06 | 1.26 | - |
| **RFCA** | 0.49 | 0.55 | 0.60 | 0.71 | 0.84 | 0.78 | 0.65 | 0.74 | 0.85 | - |
| **CBA** | 0.0 | 0.0 | 0.0 | 0.0 | 0.0 | 0.15 | 0.28 | 0.33 | 0.41 | - |

Abbreviation: AF, atrial fibrillation; AFCA, atrial fibrillation catheter ablation; CBA, cryoballoon ablation.
